# Supplementary material for: The syntax of wh-questions in unaccusative and (Un)ergative structures in Mehri language: A Phase-based approach
Source: PLoS One. 2023 Oct 12;18(10):e0289779. doi: 10.1371/journal.pone.0289779 (PMC10569537; doi:10.1371/journal.pone.0289779)
Supplement: Attachment — Submitted filename: Review PLOS ONE.pdf [file pone.0289779.s002.pdf]

Review on

"The Syntax of Wh-Questions in Unaccusative and Unergative Structures in Mehri Language: A Phase-Based Approach

This paper deals with an interesting topic on wh-questions in Mehri unaccusative and unergative constructions. However, the quality of this paper is negatively influenced by the organization of this paper, the (in-)completeness of data, and sloppy logic. It still requires substantial revision to meet the publication standard, for the following reasons:

First, much work should be done to improve the logical arrangement of the contents: (1) As a journal paper, the research question(s) should be presented clearly in the beginning, say, in the introduction part or a separate part before data analysis. However, the introduction part is devoted to a detailed description of the Melhi language, without touching upon the research questions per se. (2) Without clearly presenting the research questions, the authors conduct the literature review in a rather vague manner, ranging from wh-constructions in Arabic to the general distinction between unergatives and unaccusatives. Some of the reviews, however, are not closely related to the research questions. It may work for theses, but not for journal articles. (3) Since the research questions are not clear, the conclusions are far from being concise and convincing enough.

Second, I would like to read the answers to the following questions:

(1) What is the working mechanism for wh-questions in active constructions? Is it wh-movement to [Spec, CP] or wh-in-situ? And why?
(2) What are the patterns for wh-questions in unaccusatives? Do they follow wh-movement or wh-in-situ? And why?
(3) What are the patterns for wh-questions in unergatives? Do they follow wh-movement or wh-in-situ? And why?
(4) What are the main differences with regard to wh-questions in the unaccudatives and in the unergatives? Which one patterns more with wh-questions in active constructions? Why?

Third, in order to work out the patterns for wh-questions, I found the data provided are far from being adequate, for example:

(1) In the transitive constructions (pp5-12), the authors provided six examples. As for the wh-questions for objects, is it possible to resort to the wh-in-situ mechanism? If yes, are there any semantic differences (e.g., focus reading vs. non-focus reading) between wh-movement and wh-in-situ, given that FocP is crucial in the analysis of wh-movement? As for the wh-questions for subjects, is it possible to have wh-in-situ in the VSO patterns? I suggest that a full pattern of wh-questions for transitive constructions be well presented, before moving to wh-questions in unaccusatives and unergatives.
(2) In the unaccusatives, [PP + V + WHsubj] (see example 14 on page 23) is not acceptable. However, we don't know whether [PP + V + Subject] is acceptable. Without this information, I can hardly judge whether the authors' analysis is on the right track or not.

(3) In the unergatives, [V + WHsubj + PP] (see example 18 on page 28) is not acceptable. How about [V + WHsubj + Object] in transitive constructions?

Without adequate examples and clear patterns, the explanations cannot be sufficiently convincing.

Fourth, I have some questions regarding the following details:

(1) The accusative verb (on page 19) → Do you mean a "causative verb"?

(2) What is the "illogical nominative subject" (p 25)? Why is it illogical? Any references?
