# Supplementary material for: The syntax of wh-questions in unaccusative and (Un)ergative structures in Mehri language: A Phase-based approach
Source: PLoS One. 2023 Oct 12;18(10):e0289779. doi: 10.1371/journal.pone.0289779 (PMC10569537; doi:10.1371/journal.pone.0289779)
Supplement: Attachment — Submitted filename: Response to Reviewers.docx [file pone.0289779.s001.docx]

**Response to Reviewer #1**

1. The authors' description of Mehri seems to imply that this language is not a type of Arabic (e.g., "is threatened by the majority language, Arabic", until they later mentioned that it is a Southern Arabic language. This makes me very confused when the authors have a whole section on "wh-constructions in Arabic" which initially strikes me as irrelevant to Mehri. Thus, it is very important for the authors to be clear in the beginning that Mehri IS an Arabic language. Also, which variety of Arabic languages do the authors have in mind when comparing "Arabic" to Mehri?

The answer to comment 1 is provided in the text of the paper (See the introdcution).

As a sub-group of the Semitic language family (which is a branch of Afroasiatic family), Mehri is the most spoken of the Modern South Arabian languages (MSALs) in the present Arabian Peninsula. In other words, Mehri is a Southern Arabic language. Before the advent of Islam, Mehri and its sister MSALs were spoken largely in the southern part of the Arabian Peninsula. Moreover, traditional and modern Arabic scholars, who worked on Arabic dialects, stated that Mehri is not only a branch of the Southern Eastern Arabic language spoken in Al-Mahra region and Socotra in Yemen, but also an old Arabic tongue.

2. When mentioning the terms "unaccusative" and "unergative", please provide examples (maybe place them in brackets like e.g., 'xxxxxx'). The authors only mentioned the examples on p.11, which is too late.

The answer to comment 1 is provided in the text of the paper as an endnote. This endnote is as follows:

“Unaccusative/ergative and Unergative verbs are one-place predicate. They are traditionally called intransitive verbs. Unaccusative/ergative verbs in Mehri language are derivatives in which the external argument is not a true subject. They are derived from accusative verbs. The affixed element -**ta**- (attached to verbs) is the unaccusative marker. This can be exemplified in Table (1).

| Accusative  Two-place predicate | Unaccusative  One-place predicate | Gloss |
| --- | --- | --- |
| ***fiṩṩ***  ***ḥiṣṣ***  ***riṩṩ***  ***ķiṩṩ***  ***raḥāṩ***  ***ḥamōṩ*** | ***fataṩ***  ***ḥataṣ***  ***rataṩ***  ***ķataṩ***  ***rataḥaṩ***  ***ḥatamaṩ*** | explode  roughly tie  partly break  damage  wash  sour |

Contrastively, the unergative verbs select an external true subject that theta-marks an agent or experiencer theta-role. This can be demonstrated in Table (2):

| Unergative  One-place predicate | Gloss |
| --- | --- |
| ***šaḥrōd***  ***sayūr***  ***ḏalūf***  ***baķūṩ***  ***šawkōf***  ***śaxawlōl*** | become tired  go  jump  run  sleep  set down |

3. On p.6, reference [22] mentioned that preverbal DPs are base-generated in spec-TopP. The authors' description seems to be that subjects are not base-generated. They subsequently move to the spec-TopP. The authors did not explain this contradiction. Do the authors agree with or disagree with reference [22]?

The authors think that there is no contradiction in this assumption. Both Mehri and Arabic do have SVO and VSO. The preverbal DPs are triggered to move to the spec-TopP configuration via edge and topic features, which are inherited from C.

4. There are a lot of typos in this manuscript. For example, the glossing in (4) on p.6 is incorrect. The word order is OVS ('what ate Ahmed?'). I also see frequently "Error! Reference source not found" on p. 14-15. This causes troubles for me when I try to understand what the authors try to convey. There are other typos which I will not list here. Please correct them.

The answer to comment 4 is provided in the text of the paper. The glossing (4) on p. 6 has been corrected. “(The word order is OVS ('what ate Ahmed?')” has been corrected. "Error! Reference source not found" on pp. 14-15” has been corrected.

5. Please explain what "radical roots" means on p.13 (including the terms like "trilateral"). Some readers – especially people not working on Arabic – are not familiar with such terms.

The authors have added an endnote (in the end of the paper) that explains what “radical roots” mean. It also illustrates what the terms “trilateral roots” and quadrilateral roots” mean. This endnote is as follows:

“In Semitic languages, the verb roots are indicated by a sequence of consonants (dubbed as radicals); this invites the terminology ‘consonantal root.’ In Arabic, for instance, consonantal roots are used to form words by the addition of vowels and non-root consonants. There also are other terms used in Arabic such as, “trilateral roots” and quadrilateral roots”. A trilateral root (dubbed as ʻtriconsonantal rootʻ) is viewed by Arab and Western scholars as a root that has a sequence of three consonants (e.g., [k-t-b](https://en.wikipedia.org/wiki/K-T-B)  ك-ت-ب "to write"). A quadrilateral root, on the other hand, is a consonantal root that has a sequence of four consonants. A quadriliteral form is a word derived from four consonantal roots in Arabic (e.g, the quadriliteral root عرقل ʻto obstructʻ).”

6. The figures look very crowded. Please make the pictures bigger and tidier. When I read the detailed descriptions, I sometimes found it easier to simply follow the text than check the figure.

The authors have made the tree-diagrams bigger and tidier in the text of the paper; we have changed those which are not clear and have also enlarged the small ones.

7. Although the authors do not aim to provide unexpected data to make a new point for the field, it would be nice if the authors could mention or add some implications of their analysis for the field, other than simply confirming the established phase theory. If a linguist not working on this language reads this paper, what do they gain?

The answer to comment 7 is provided in the conclusion of the paper. The authors have modified the conclusion and have also added some implications of the study. This can be demonstrated in the end of the conclusion section.

Furthermore, the study provides theoretical implications; the findings of the study provide further support to Chomsky’s Phase Theory, where the analysis has shown that Mehri obeys the Phase Impenetrability Condition proposed in [2], which assumes that when all syntactic operations in a given phase have been achieved, the domain of that phase becomes impenetrable to any syntactic operation in the syntax.

Another implication is that, unlike the assumption of [4449], which states that ergativity in Aramaic dialects is unique in Semitic tradition due to the contact with ergative languages such as Kurdish and Iranian languages, this article demonstrated that the ergative morphology is invariably attested in Mehri, which belongs to the Semitic language family. It is also hoped that the syntactic findings of the study will contribute to the understanding of the syntax of wh-questions in the Semitic language family and to the existing literature on the Arabic language and its varieties as well as on other languages in the world.

**Response to Reviewer #2**

“First of all, the manuscript should be carefully proofread before future re-submission. Currently, it is filled with missing/wrong references to examples and figures. For example, there are more than one figures labeled figure 1, same with figure 2; on page 7 just below figure 1, there is an in-text figure reference without any numbering; on pages 14-16, there is something wrong with example referencing (it reads “Error! Reference source no found” in text several times); on page 15, there are multiple references to a non-existing example “0”. In addition to all these errors, there are also a lot of typos, e.g. the word “noble” should be “novel” in the abstract, the word order in the gloss of example (4) is wrong, there is a sentence towards the middle of page 18: “… remains in-situ in.” which is either unfinished, or contains an extra “in”, etc.. All these problems are extremely distracting, and make the manuscript hard to read.

The paper has been proofread. The missing/wrong references to examples and figures have been checked and corrected accordingly in the text of the paper. “The word “noble” should be “novel” in the abstract” has been corrected. “The word order in the gloss of example (4)” has been corrected. “There is a sentence towards the middle of page 18: “… remains in-situ in.” which is either unfinished, or contains an extra “in”, this sentence has been corrected in the text of the paper.

The second reviewer stated that “on pages 14-16, there is something wrong with example referencing (it reads “Error! Reference source no found” in text several times); on page 15, there are multiple references to a non-existing example “0.”

"Error! Reference source not found" is corrected everywhere in the manuscript.

Concerning this comment "there are multiple references to a non-existing example “0" has been corrected.

- Section 1: This is a rather lengthy discussion on wh-movement in Arabic. This whole discussion has very little to do with how wh-movement works in Mehri, which I assume is the main point of this paper. Just because Arabic and Mehri are related languages does not mean that the authors can make syntactic assumptions about Mehri based on what has been established in Arabic. I recommend either removing this discussion entirely, or make clear what this discussion has to do with the rest of the paper.

The answer to comment 1 is provided in the text of the paper. The authors have made clear what this discussion on wh-movement in Arabic has to do with the rest of the paper.

They have provided some justifications. Please see the beginning of that section on wh-movement in Arabic.

This section reviews the previous analyses conducted by Arab scholars on wh-questions in Arabic syntax. The reasons why this section begins reviewing the previous analysis of wh-questions in Arabic can be attributed to the following: (i) both Mehri and Arabic belong to the Semitic language family, which also includes Aramaic, Amharic, Syriac, Canaanite, and southern Arabian languages- one of which is Mehri [13]. (ii) Both Mehri and Arabic exhibit two prominent word orders: SVO and VSO. Apart from Arabic that exhibits agreement asymmetry between a verb and its post-verbal subject within VSO order, Mehri appears with a full agreement in both SVO and VSO word orders [12]. (iii) Both Mehri and Arabic exhibit certain syntactic properties with regard to wh-movement in unaccusative and (un)ergative structures. The analysis of Arabic wh-questions reviewed in this subsection will facilitate the understanding of the syntactic discussion of wh-questions in Mehri’s unaccusative and (un)ergative structures. Moreover, it is true that Arabic and Mehri indeed are related languages, but it does not mean that the syntactic assumptions in the present study about Mehri will be based on what has been established in Arabic. What distinguishes the analysis adopted in this study from those on Arabic wh-questions reviewed below is that the analysis about Mehri is based on the Phase Theory of Chomsky (2008)[2], whereas the previous accounts on Arabic wh-question discussed below adopted the Government and Binding theory and early minimalist frameworks. Consequently, the approaches of the analyses are different and the findings are dissimilar.

- p.12: I recommend having a tree demonstrating the split-CP hypothesis, which is implicitly adopted throughout the manuscript (the ForceP-FocP-TopP left periphery). This is worth making clear, since the ordering of FocP and TopP is doing a lot of the heavy-lifting in accounting for examples like (11) and (12) later on.

The authors have added a sub-section on Rizzi’s (1997) Split-CP Hypothesis illustrated by Rizz’s Tree-diagram immediately after the Phase Theory framework section.

Rizzi’s [35] Split-CP Hypothesis

In this sub-section, we introduce Rizzi’s [35] seminal analysis of the fine structure of CP. The objective of introducing Rizzi’s Split-CP is that parts of the syntactic analysis of the present study adopted Rizzi’s proposal, especially TopP and FocP projections.

A closer look at the traditional understanding of the clause reveals that it consists of three syntactic layers: CP, IP, and VP. As the CP indicates a speaker’s attitude, the IP illustrates grammatical input (tense and agreement features). The VP, on the other hand, contains arguments. Each of these layers has been split into separate projections; the CP is split in [35], the IP is in [36], and the VP in [37]. In the last two decades, [35], [38], and [39], to name a few, proposed a syntactic expansion of a functional projection in order to accommodate the complementizer (C) and other syntactically relevant material existing on the clause left periphery. [35] suggested that the CP projection of a clause structure should be split into a number of separate functional projections in the syntax in order to host elements that surface on the clause's left periphery. In Rizzi’s [35] analysis, a head complementizer (C) was viewed as a Force marker, thus heading Force Phrases. Furthermore, topicalized and focused constituents were viewed as different Topic Phrases (headed by Top) and Focus Phrases (headed by Foc). Hence, the outcome of the proposal is that there are separate projections, each one having a specifier position and a head-on clause structure. Rizzi’s Split-CP structure can be illustrated below in Figure 4.

**Figure 4. Tree structure of Rizziʻs [35] Split-CP Hypothesis**

A closer look at Rizzi’s hierarchical ordering of the expanded functional projections reveals that the projection ForceP dominates TopP, which dominates FocP, which dominates FinP; this would look like (… Force … (TopP) … (Foc) … FinIP). Moreover, [38] modified the preceding proposal, where he added IntP (interrogative projection) to the clause structure to account for wh-questions. It has been demonstrated in the existing literature that in Germanic languages topics undergo V-movement, whereas they do not in English; in the latter (English), the head of the Topic does not need to be filled in the syntax.

- Middle of p.13: “Chomsky stressed that when all syntactic operations in a given phase have been completed…” Merger of the phase head triggers the spell out of phasal complement. Merger of phase head is crucially different from “when all syntactic operations in a phase are completed”.

These two clauses have been deleted.

- Bottom of p.17: “Meanwhile, the lexical V ‘exploded’ raises to the head T position in order to …” You haven’t yet established whether/how V-to-T head movement in Mehri works (observing that it happens in Arabic is not a justification that the same happens in Mehri). Please provide justification that V-to-T movement happens in this case, or state clearly that this is an assumption you are making (rather than stating it as part of your proposal).

In Mehri language, all lexical verbs must combine temporal and agreement features. These features are valued through embedded affixes within a verb, or sometimes through melodies as demonstrated in the lexical V ***fataṩṩ*** ‘exploded’. In contrast to the V ***fataṩūt*** ‘exploded’ that combines perfective and agreement (i.e., 3fs) features, the lexical V ***fataṩṩ*** ‘exploded’ semantically bears perfective and agreement (i.e., 3fp) features. T, on the other hand, bears the same features but they are unvalued. It, then, probes down searching for a goal or a checker. The only goal available is the lexical V ***fataṩṩ*** ‘exploded’. It covertly joins T to constitute a verbal complex (T+V).

- Bottom of p. 17: “… it can be observed in the preceding examples (8) and (9) that the head T contains unvalued agreement features and an EPP feature.” How do (8) and (9) show you that T has an EPP feature? (8) and (9) only shows that the subject DP precedes the V. How do you know that the subject DPs are in Spec-TP (as opposed to, for example, Spec-TopicP)?

The finite declarative/interrogative force of C inherits formal features (i.e., agreement, temporal and case) to T. To verify these features, T probes down searching within VP for a goal. This goal should be *aśxūf* ‘the milk’ in (7) and *anīʕāytan ḏa-ḥāyabīt* ‘the camel’s breasts’ in (8-9). In (7), the external subject *aśxūf* ‘the milk’ must agree with a hybrid complex accusative V *fiṩṩ* ‘explode’ in agreement features (i.e., masculine, person & 3^rd^ number) while the external subject *anīʕāytan ḏa-ḥāyabīt* ‘the camel’s breasts’ in (8-9) must agree with a hybrid complex unaccusative V *fataṩṩ* ‘explode’ in agreement features (i.e., feminine, plural & 3^rd^ number) (see alqumairi and Shuib 2018). On the other hand, the internal object *anīʕāytan ḏa-ḥāyabīt* ‘the camel’s breasts’ in (7) covertly bears an accusative case feature. After triggering movement to Spec-TP in (8-9), the extracted unreal subject changes its original case to a nominative. This actually occurs via an Extended Projection Principle (EPP) of T that requires a subject of TP. Let us consider the following examples to illustrate the point.

10. fataṩṩ	man aśxūf.

[explode-3fp.Perf]	from the-milk

‘They exploded from the milk.’

11. aśxūf	fiṩṩaysan.

The milk explode-3ms-them

‘The milk exploded them.’

Evidently, Mehri is a pro-drop language. In (10), the external subject is lexically dropped while its formal features within T are still associated with unaccusative V *fataṩṩ* ‘explode’. In contrast, the clitic *-aysan* in (11) indicates an internal accusative object *anīʕāytan ḏa-ḥāyabīt*.

- More generally, how do we know that (9) is an instance of overt wh-movement, rather than topicalization of an in-situ wh-PP? Or maybe just base-generation of the wh-PP at a high position? One idea is to test whether this movement is island sensitive (and if so, that rules out the base-generation possibility). You will still need some independent evidence to rule out the topicalization possibility.

The wh-PP is an adjunct that is replaced by the PP ***man-*** *aśxūf.* This wh-PP receives high pitch from the speaker. It is focalized and moved to Spec-FocP. As for the evidence, let us consider a parallel example:

hibōh  ķatal  aśxūf?

‘How did the milk throw down?’

aśxūf ķatal  **baḥays**.

Or **baḥays**, aśxūf ķatal

‘The milk strongly threw down’

The wh-advP ***baḥays*** ‘strongly’ is focalized and moved to the Spec-FocP configuration.

- p. 18: “From Mehri examples (8-9), the head T inherits tense, case, and edge features from C…” Please be clear how the two sentences in (8) and (9) show this (especially, that T inherits EF from C).

The finite declarative/interrogative force of C inherits formal features (i.e., agreement, temporal and case) to T. To verify these features, T probes down searching within VP for a goal. This goal should be *aśxūf* ‘the milk’ in (7) and *anīʕāytan ḏa-ḥāyabīt* ‘the camel’s breasts’ in (8-9). In (7), the external subject *aśxūf* ‘the milk’ must agree with a hybrid complex accusative V *fiṩṩ* ‘explode’ in agreement features (i.e., masculine, person & 3^rd^ number), while the external subject *anīʕāytan ḏa-ḥāyabīt* ‘the camel’s breasts’ in (8-9) must agree with a hybrid complex unaccusative V *fataṩṩ* ‘explode’ in agreement features (i.e., feminine, plural & 3^rd^ number) (see Alqumairi and Shuib 2018). On the other hand, the internal object *anīʕāytan ḏa-ḥāyabīt* ‘the camel’s breasts’ in (7) covertly bears an accusative case feature. After triggering movement to Spec-TP in (8-9), the extracted unreal subject changes its original case to a nominative. This actually occurs via an Extended Projection Principle (EPP) of T that requires a subject of TP. To illustrate the point let us consider the following examples:

10. fataṩṩ	man aśxūf.

[explode.3fp.Perf] from	the milk

‘They exploded from the milk.’

11. aśxūf  fiṩṩaysan

The.milk explode.3ms-them

‘The milk exploded them.’

Evidently, Mehri is a pro-drop language. In (10), the external subject is lexically dropped while its formal features of within T are still associated with unaccusative V *fataṩṩ* ‘explode’. In contrast, the clitic -*aysan* in (11) indicates an internal accusative object *anīʕāytan ḏa-ḥāyabīt* ‘the camel’s breasts.’

- Top of p.19 “Since this DP is definite and is located in Spec-TopP, it can be assumed as a Topic”. Why is it in Spec-TopP, as opposed to Spec-TP? No independent evidence is provided. Some diagnostics for structural height could be helpful here (e.g. ordering between the DP and certain TP-adjuncts). - bottom of p.19: “… it is triggered to move to Spec-TopP because it is definite.” Why would definiteness trigger movement to Spec-TopP in Mehri? Right now the wording makes it sounds like all definite DPs in Mehri need to undergo Topic movement (which can’t be right). Is there independent evidence for this?

According to Watson (2012) and Rubin (2010), Mehri exhibits (in)definiteness. The definite markers in Mehri are always prefixed by *a-* and *ha-* such as, *arawram* ‘the sea’ *rawram* ‘a sea’, *hayabīt* ‘the camel’ *yabīt* ‘a camel’ *yabīt* ‘a camel’, while the indefinite marker is null. Semantically, with the definite articles, the speakers always signal to the hearer that the referent of the DPs is familiar to both interlocutors. However, let us consider the following examples:

A). anīʕāytan ḏa-hayabīt  lyakmah  fataṩṩ	man	aśxūf.

The camel’s breasts(def.3pf.) those(3pf) explode(erg.perf.3pf.) from the-milk

‘Those camel’s breasts exploded from the milk.’

B). nīʕāytan ḏa-yabīt	fataṩṩ	man	aśxūf.

camel’s breasts (indef. 3pf.) explode(erg.perf.3pf.) from	the-milk

‘Camel’s breasts exploded from the milk.’

C). *nīʕāytan ḏa-yabīt lyakmah	fataṩṩ	man aśxūf.

camel’s breasts(indef.3pf.) those(3pf)	explode(erg.perf.3pf.) from the-milk

The example in (A) shows the situational use of the definiteness. The referent of the DP *anīʕāytan ḏa-hayabīt* “the camel’s breasts” is familiar to the interlocutors through a physical situation, which contributes this familiarity. In other words, the hearer can topicalize and easily pick out the intended referent because it is familiar to him from an immediate and visible situation. In (B), however, we have an example of unfamiliar use of the indefinite DP *nīʕāytan ḏa-yabīt* ‘camel’s breasts’, its referent is not familiar to the hearer from a visible situation. In contrast, the example in (C) is not acceptable because the indefinite DP cannot be associated with a definite demonstrative article such as, *lyakmah* ‘those.’ Let us consider the following tree-diagram to illustrate the point.

As demonstrated in the above clause structure of Mehri, the DP *anīʕāytan ḏa-hayabīt lyakmah ‘those camel’s breasts’* is gradually moved from the Spec-VP projection to Spec-TP and then to Spec-TopP.

- Is there independent evidence that wh-DPs can be licensed in-situ in Mehri?

The evidence that wh-DPs can be licensed in-situ in Mehri can be demonstrated in the following example and its clause structure.

haśan	laykmah	fataṩṩ	man	aśxūf?

what	those(3pf)	explode (erg. perf. 3pf.) from	the-milk

‘What (those) exploded from the milk.’

From the example above and its syntactic representation in the clause structure, the topicalized DP *anīʕāytan ḏa-hayabīt ‘the camel’s breasts’* is replaced by wh-word *haśan ‘what’* and is moved to the Spec-ForceP configuration, while the demonstrative article *laykmah* ‘those’ occupies Spec-TopP.

Moreover, in Mehri language, two types of questions can be licensed: fronted wh-questions and in-situ wh-questions. According the existence of formal features (i.e., EF, Topic and Focus) within a clause, the following syntactic options will be constructed:

1. When the external DPs are definite, the wh-subject will move tp the spec-TopP configuration.

2. When the external DPs are indefinite, the wh-subject will remain in-situ in the Spec-TP configuration.

3. When the internal object or adjunct can be moved to the Spec-FocP configuration via an edge feature inherited from C (i.e., Force), otherwise it will remain in-situ.

- p.21: traditionally, experiencer arguments are thought to be VP-internal arguments (Landau 2010). Under this view, wouldn’t “become tired” actually be projecting “camel” VP-internally unlike an unergative verb? Why not just use clearly unergative verbs like “walk” or “swim” in your analysis instead?

Similar to various unergative verbs (i.e., *sōbaḥ* ‘swim’, *baķōṩ* ‘run’, *šawkōf* ‘sleep’ etc.), the unergative verb ***tašaḥrawd*** ‘become tired’ only projects one argument. This argument must be a pre-verbal or post-verbal subject that theta-marks an experiencer or agent. We use the *ša-*type verbs such as ***tašaḥrawd*** ‘become tired; 3fs’ or ***šaḥrawd*** ‘become tired; 3ms’ only to represent the derived unergative verbs in Mehri language.

- middle of p.25 “… because the temporal complex is banned from crossing over the wh-word…” Why is V-to-T movement blocked by the wh-word? There is no explanation provided.

V-to-T movement is blocked by the wh-word because the wh-word replaces the external subject ***ḥāybīt ḥōfay*** ‘the milked camel’.

- Is the unergative counterpart of example (11) possible? Under your analysis, if the adjunct “man masyer” is moved to Spec-Foc, the wh-DP could be licensed in-situ (maybe in Spec-TopP) with only covert movement of a Q-feature. This predicts a sentence like “man masyer haṡan tašaḥrawd” to be possible.

Yes, the unergative counterpart of example (11) is possible in Mehri.

- What about multiple-wh questions (e.g. Who ran to which house)? Is that a possibility in Mehri? Either way, it would be a nice testing bed for the proposed theory.

Yes, Mehri permits wh- and multi wh-word movement. This can be illustrated in the examples below.

- mūn	baķūṩ	lhal  haśan	man bayt?

Who run (perf.3ms.) to	which	of	house

‘Who ran to which house’

- mūn	katūb haśan?

Who write(perf.3ms.) what

‘Who wrote what?’

- wakōh hẽt	atbayk	mūn	sabṫaw-k?

Why	you cry (prog.2ms.) who	hit (imperf.3ms.)-you

‘Why are you crying? Who hit you?’

- wakōh hẽt ḏašḥradk, damlak	haśan?

Why	you tired(perf.2ms.) do(perf.2ms.) what

‘Why did you become tired, what did you do?
