# Supplementary material for: The syntax of wh-questions in unaccusative and (Un)ergative structures in Mehri language: A Phase-based approach
Source: PLoS One. 2023 Oct 12;18(10):e0289779. doi: 10.1371/journal.pone.0289779 (PMC10569537; doi:10.1371/journal.pone.0289779)
Supplement: Attachment — Submitted filename: Response to Reviewer.docx [file pone.0289779.s003.docx]

**Response to Reviewer #1+3**

Additional Editor Comments:

Thank you for your valuable edits.

There are a few things to be worked on (specifically when it comes to “the literature lacks of” because there are studies on the matter) & I think the authors can refine their debate.

The authors modified and refined the debate of “the literature lacks of” in the first and second rounds of the comments suggested by the editor and the reviewers. In this third round of paper editing, again, the authors made some modifications and changes in various places in the paper in response to this comment (kindly see the following pages: abstract, p. 12, and p. 18). Moreover, it is true that there are studies on the matter but such studies explored and analysed the syntax of wh-questions in the world languages, but not the syntax of wh-questions of unaccusative and (un)ergative structures in Mehri within minimalism.

Please carefully address reviewers’ comments, and the new one - since a previous reviewer was not able due to workload. These comments are really interesting too, so both reviewers’ criticisms need to be considered.

6. Review Comments to the Author

Please use the space provided to explain your answers to the questions above. You may also include additional comments for the author, including concerns about dual publication, research ethics, or publication ethics. (Please upload your review as an attachment if it exceeds 20,000 characters)

Reviewer #2:

Thank you for the responses to my previous comments and questions.

You are welcome.

This draft is ready for publication with some very minor revisions:

Thank you so much.

- For example, on p.12, [Spec, vP] is incorrectly spelled as [Sec, vP].

The authors have corrected “[Sec, vP]” to [Spec-vP]” on page 12.

- On p.13, "d-structure" should be "D-structure", following the notational tradition in syntax.

The authors have corrected “[d-structure]” to “D-structure” on page 13.

- In Figure 4, please be clear what the stars on the two TopP projections represent. Also, Rizzi used IP instead of TP in his notation, but this manuscript uses TP throughout. Please make sure it is consistent, and maybe change the IP to TP in Figure 4 to reduce confusion by readers.

1. Concerning the stars on the two TopP projections, in fact, the authors have cited the same clause structure proposed by Rizzi (1997), where the original author (Rizzi) used these stars in a paper titled *"Rizzi, Luigi (1997). The fine structure of the left periphery. In L. Haegeman. (ed.), Elements of Grammar. Dordrecht: Kluwer.*” The authors think that it is not logical to change the original clause structure of the original author without his consent. We suggest that it can be cited as it is. Moreover, Rizzi’s (1997) article has been cited by linguists and research scholars, who used the same clause structure with stars on the two TopP projections.

2. The same thing applies to the IP projection in Figure 4. The authors think that it would be better if you kindly allow them to leave the IP unchanged, and keep it as it is proposed in Rizzi’s (1997) clause structure illustrated in Figure 4. Ethically, we feel that it is better if we abide by the copyrights of the original author.

Moreover, by the advent of minimalism in the 1990s onwards, the TP projection was largely used in the minimalist syntax. The syntactic use of TP has wider scope than that of IP. That is why TP has commonly been used in syntactic theory.

- Rizzi has two TopP projections, a high one above FocP, and a low one below FocP. You are only making use of the high one (as shown in Figure 8). Please explicitly state this choice, and provide motivations if you can.

The EPP attracts the external subject from the [Spec-v*P] projection to the Spec-TP] projection. It is then moved to [Spec-TopP] via an EF inherited form CP (ForceP). Kindly, have a look at the following clause structure, where the two projections of TopP are illustrated.

- On p.17, the "FinIP" in "(… Force … (TopP) … (Foc) … FinIP)" should be FinP

On page 17, the authors have corrected the "FinIP" in "(… Force … (TopP) … (Foc) … FinIP)" into FinP

- Sometimes the authors use the notation [Spec, XP], and sometimes they use [Spec-XP]. Please be consistent.

The authors have corrected the notation “[Spec, XP]” into [Spec-XP] throughout the study. The notation [Spec-XP] is now unified and consistent throughout the paper.

- Bottom of p.30: v*p should be v*P

The authors have corrected “v*p” into v*P in the bottom of page 30.

.

.

.

.

.

Reviewer #3:

This paper deals with an interesting topic on wh-questions in Mehri unaccusative and unergative constructions. However, the quality of this paper is negatively influenced by the organization of this paper and the (in-)completeness of data. It still requires substantial revision to meet the publication standard, for the following reasons:

First, much work should be done to improve the logical arrangement of the contents: (1) As a journal paper, the research question(s) should be presented clearly in the beginning, say, in the introduction part or a separate part before data analysis. However, the introduction part is devoted to a detailed description of the Melhi language, without touching upon the research questions per se. (2) Without clearly presenting the research questions, the authors conduct the literature review in a rather vague manner, ranging from wh-constructions in Arabic to the general distinction between unergatives and unaccusatives. Some of the reviews, however, are not closely related to the research questions. It may work for theses, but not for journal articles. (3) Since the research questions are not clear, the conclusions are far from being concise and convincing enough.

1. In response to the comment of Reviewer #3, the authors have modified the introduction, where they have presented clearly the research questions of the study on page 5, as follows: “The study seeks to answer the following research questions within Chomsky’s [1.2] Phase-based Theory: (i) Does Mehri language allow fronting of wh-phrases to [Spec-CP]? and (ii) how can wh-question movement in Mehri unaccusative and (un)ergative structures be accounted for?”

## 2. On page 18, in the section of Mehri analysis, and under the heading “Analysis of wh-questions in (un)accusative/ergative structures in Mehri”, the authors have incorporated the research questions there, as follows:

“Compared to other endangered languages, not much attention has been paid to the syntax of wh-questions in Mehri. The study aims to provide an adequate answer to the following research questions: (i) Does Mehri language allow fronting of wh-phrases to [Spec-CP]? and (ii) how can wh-question movement in Mehri unaccusative and (un)ergative structures be accounted for neatly within Chomsky’s [1.2] Phase-based Theory?”

3. The conclusion of the study is also modified based on the inclusion of the research questions. This can be seen on page 35.

“Moreover, the study presented an adequate answer to the research questions; it explained that Mehri language allows fronting of wh-phrases to [Spec-CP]? and provided a satisfactory account of wh-question movement in Mehri unaccusative and (un)ergative structures within Chomsky Phase-based Theory.” (What follows these lines also summarizes the answer of the study questions."

Second, I would like to read the answers to the following questions:

(1) What is the working mechanism for wh-questions in active constructions? Is it wh-movement to [Spec, CP] or wh-in-situ? And why?

Mehri exhibits two mechanisms for wh-questions: overt wh-question movement and wh-in-situ questions. Let us consider the following examples (a-c) to illustrate the point.

a. Ali  tïķ	ḥamōh	SVO

Ali drink	the-water

‘Ali drank the water.’

b. Ali tïķ  **haśan**?

Ali drank **what**

c. **haśan** Ali	tïķ?

**What**	Ali	drink.

‘What did Ali drink?’

The examples in (b) and (c) are active constructions in SVO word order, which show wh-questions. In (b), the wh-object word ***haśan*** ‘what’ remains in-situ; it replaces the object NP *ḥamōh* 'the water.' In (c), on the other hand, the wh-object word ***haśan*** ‘what’ undergoes overt movement to the left periphery of the clause; it moves to the [Spec-CP] projection via the strong wh-feature of the head C.

(2) What are the patterns for wh-questions in unaccusatives? Do they follow wh-movement or wh-in-situ? And why?

The patterns for wh-questions in unaccusatives in Mehri can be illustrated below:

a. ḥamōh  katal	baķa.

The-water	spille out	on the ground

‘The water spilled out on the ground.’

b. **haśan** katal?

What	spilled out

'What spilled out?'

c. **hōh**	katal	ḥamōh?

Where	spilled out	the water

‘Where did the water spill out?’

d. ḥamōh	katal	**hōh**?

the-water spilled out	where

‘Where did the water spill out?’

e. ḥamōh  **hōh**  katal?

the-water where spilled out

‘Where did the water spill out?’

f. katal	**hōh**	ḥamōh?

spilled out	where the-water

‘Where did the water spill out?’

As for the wh-subject, the definitive subject must be replaced by a wh-word and then is moved overtly to the [Spec-TopP] configuration, while the indefinite subject must remain in-situ in [Spec-TP]. Given wh-object, three patterns can be presented: the wh-movement as in (c) via focalization, the in-situ question as in (d), which is not focalized, and the partial wh-movement (e) and (f) via partial focalization.

(3) What are the patterns for wh-questions in unergatives? Do they follow wh-movement or wh-in-situ? And why?

The patterns of the wh-questions in unergatives in Mehri can be demonstrated below.

a. Ĝagnūt šawkfūt brak aṩayga

the-girl slept	in	the-cave

‘The girl slept in the cave.’

b. **mūn**  šawkfūt brak aṩayga?

who	slept	in	the-cave

‘Who slept in the cave?’

c. **hōh**  šawkfūt	Ĝagnūt?

where	slept	the-girl

‘Where did the girl sleep?’

d. Ĝagnūt	šawkfūt **hōh**?

the-girl slept	where

‘Where did the girl sleep?’

e. Ĝagnūt **hōh**  šawkfūt?

the-girl where slept

‘Where did the girl sleep?’

f. šawkfūt **hōh** Ĝagnūt?

slept	where the-girl

‘Where did the girl sleep?’

The (V)erb √škf “to sleep” is intransitive, specifically, it is an unergative type that assigns one argument. It is an experiencer subject. In (a), the V *šawkfūt ‘slept’* assigns an experiencer subject NP, *Ĝagnūt* ‘the girl’, while the PP *brak aṩayga* ‘in the cave’ is an optional locative goal that doesn’t affect the meaning if it deleted. Based on the (in)definiteness, the wh-subject can either move to [Spec-TopP] (see example (b)) or to [Spec-TP]. As for the wh-object question, the wh-word undergoes movement to the left periphery of the clause via the head Foc and EF inherited from ForceP. However, three patterns can be observed: the front (overt) wh-movement as in (C), the in-situ pattern, which is not focalized, as in (d), and the partial wh-movement as in (e-f).

(4) What are the main differences with regard to wh-questions in the unaccusatives and in the unergatives? Which one patterns more with wh-questions in active constructions? Why?

Based on our understanding of Mehri and on the illustrative examples above, the authors observe that there are no differences with regard to wh-questions in the unaccusatives and in the unergatives in Mehri, because both are intransitive types, which need to assign only one argument NP.

Third, in order to work out the patterns for wh-questions, I found the data provided are far from being adequate, for example: (1) In the transitive constructions (pp5-12), the authors provided six examples. As for the wh-questions for objects, is it possible to resort to the wh-in-situ mechanism? If yes, are there any semantic differences (e.g., focus reading vs. non-focus reading) between wh-movement and wh-in-situ, given that FocP is crucial in the analysis of wh-movement?

To illustrate this question, let us consider the following examples of Mehri:

a. aĝayg	ṯabūr	axalfẽt.

the-man broke	the-window

‘The man broke the window.’

b. aĝayg	ṯabūr	**haśan**?

the-man broke	what

‘What did the man break?’

c. **haśan**	aĝayg	ṯabūr?

what	the-man	broke

‘What did the man break?’

Based on the Mehri examples in (a-c), the authors observe that the answer to this question is “Yes”. Yes, it is possible to resort the wh-in-situ mechanism as demonstrated in (b). Moreover, there are no semantic differences between (b) and (c). However, we assume that wh-movement is the result of the strong wh-feature that is represented by either topicalization or focalization. The wh-word remains in-situ via the existence of the weak wh-feature. However, it lexically stays in-situ while the interrogative force determines the semantic reading of the clause.

As for the wh-questions for subjects, is it possible to have wh-in-situ in the VSO patterns? I suggest that a full pattern of wh-questions for transitive constructions be well presented, before moving to wh-questions in unaccusatives and unergatives.

a. aĝayg  ṯabūr  ḍïmah xalfẽt. **SVO**

the-man	broke	this window

‘The man broke this window.’

b. **mūn**	ṯabūr ḍïmah xalfẽt? **SVO**

who	broke this window

‘Who broke this window?’

c. ḍïmah xalfẽt **mūn**	ṯabrïs? Topicalized

this window	who	broke

(This window, who broke?)

d. ṯabūr	aĝayg	ḍïmah xalfẽt. **VSO**

broke the-man this window

‘The man broke this window.’

e. *ṯabūr **mūn** ḍïmah xalfẽt. **VSO**

broke who this window

f. *ḍïmah xalfẽt ṯabrïs **mūn**? **VSO**

this window broke who

The transitive V ṯabūr ‘broke’ selects two arguments in (a); the pre- or post- verbal subject, *aĝayg* ‘the man’ and the internal object, *ḍïmah xalfẽt* ‘this window’. In the SVO structure, the preverbal subject is replaced by wh-subject ***mūn*** ‘what’, as shown in (c). This is then topicalized and moved to the [Spec-TopP] projection.

As for the VSO structure in (d) (which is declarative), the post-verbal subject cannot be replaced by wh-word ***mūn***' who’. This entails that wh-in-situ mechanism is not available in VSO structure. This is evidently shown in (e) and (f).

(1) In the unaccusatives, [PP + V + WHsubj] (see example 14 on page 23) is not acceptable. However, we don’t know whether [PP + V + Subject] is acceptable. Without this information, I can hardly judge whether the authors’ analysis is on the right track or not.

Let us consider the following examples (14) on page 23 in order to explain the point clearly.

(14) **********man-****aṡxōf*	***fataṩṩ***	***haṡan?***

[from-the-milk.3ms.nom.] [exploded-fp.Perf.] what?

Languages exhibit interesting syntactic phenomena, Mehri is no exception. For example, the structure in (14) is not acceptable not Mehri grammar. The adjunct PP ***man-****aṡxōf* ‘from the milk’ is not allowed to precede the unaccusatives in Mehri language. .

(3) In the unergatives, [V + WHsubj + PP] (see example 18 on page 28) is not acceptable. How about [V + WHsubj + Object] in transitive constructions?

Without adequate examples and clear patterns, the explanations cannot be sufficiently convincing.

In response to question (3) above, let us have a closer look at the following example in (18), on page 28, to illustrate the point.

(18) **tašaḥrawd*	***haṡan***	*(man masyer)?*

[become-3fs.subj]	what	(from the walk)

In (18), the unergative verb has been intonated more than the wh-subject. This makes the interrogative clause lose its question force. Therefore, the unergatives [V + WHsubj + PP] is not acceptable in Mehri.

Moreover, the pattern [V + WHsubj + Object is not acceptable in transitive constructions in Mehri. The following example illustrates the point.

a. *ṯabūr  **mūn** axalfẽt?

broke who	the window

‘Who broke the window?’

In (a), the wh-word ***mūn*** ‘who’ replaces the post-verbal subject in the VSO structure. To construct a correct wh-subject question, the post-verbal subject must subsequently move to the [Spec-TP] projection, until reaching the final locus in [Spec-TopP]. Moreover, the extracted subject could be substituted by the wh-word ***mūn*** ‘who’ and moves to [Spec-ForceP], which results in an interrogative force.

Fourth, I have some questions regarding the following details:

(1) The accusative verb (on page 19) ***faṩṩ*** Do you mean a “causative verb”?

No, it is not a causative verb. It is an accusative verb that selects two arguments: the external argument (i.e., subject) and internal argument (i.e., object). The V ***faṩṩ*** ‘exploded’ assigns the external argument *aṡxōf* ***‘the milk’*** and internal argument ***anīʕāytan ḏa-ḥāyabīt*** ‘the camel’s breasts.’ On the other hand, the unaccusative V ***fataṩṩ*** ‘exploded’ assigns one argument, it is the external argument ***anīʕāytan ḏa-ḥāyabīt*** ‘the camel’s breasts.’ In contrast, the causative verbs in Mehri are marked by the suffix *ha-* as shown in following examples:

a. aĝayg  habhōl  ḥïrẽz  baśïwūṫ

the-man matured	rice	with fire

‘The man matured the rice with fire.’

b. ḥïrẽz  bahẽl

the-rice	matured

‘The rice matured.

In (a) the causative V *habhōl* ‘matured’ (which is derived from the intransitive V *bahẽl* ‘matured’ shown in (b)) assigns three arguments: the subjective agent *aĝayg* ‘the man,’ the thematic object *ḥïrẽz* ‘the rice,’ and the instrumented object *baśïwūṫ* ‘by fire’.

(2) What is the “illogical nominative subject” (p 25)? Why is it illogical? Any references?

Case assignment in Mehri is not marked. Unlike Standard Arabic, nominative subject and accusative object in Mehri are syntactically determined. This means they can be recognised through their syntactic locations in a clause. According to Jubilado (2021), the unaccusative/ ergative verbs in Philippine languages have only one argument. The one available argument is the one assigned a theme theta role. Similarly, the unmarked nominative subject for the unaccusative/ ergative verbs in Mehri assign only one argument that theta-marks a theme theta role. This argument is not a real subject. It actually works as the internal object for the transitive verbs that bears an accusative case. After the morphological changes to the verbs, this accusative object is extracted to move the left periphery of the clause, in which its case is changed to a nominative. However, we consider it as an illogical nominative subject. ~~See~~ The following examples can explain the point clearly.

a. aĝayg	ṣikk  axalfẽt

the-man closed	the -window

‘The man closed the window.’

b. axalfẽt	ṣatakūt

the-window	closed

‘The window closed.’

**References**

**Jubilado, R. (2021). Comparative Ergative and Accusative Structures in Three Philippine Languages. South-eastern Philippines *Journal of Research and Development*, Vol. 26, No. 1.**
