# Supplementary material for: The syntax of wh-questions in unaccusative and (Un)ergative structures in Mehri language: A Phase-based approach
Source: PLoS One. 2023 Oct 12;18(10):e0289779. doi: 10.1371/journal.pone.0289779 (PMC10569537; doi:10.1371/journal.pone.0289779)
Supplement: Attachment — Submitted filename: Response to Reviewers.docx [file pone.0289779.s004.docx]

**Responses to reviewers’ Comments**

PONE-D-23-09811R2

The Syntax of Wh-Questions in Unaccusative and (Un)Ergative Structures in Mehri Language: A Phase-Based Approach

PLOS ONE

Dear Dr. Alzubi,

Thank you for submitting your manuscript to PLOS ONE. After careful consideration, we feel that it has merit but does not fully meet PLOS ONE’s publication criteria as it currently stands. Therefore, we invite you to submit a revised version of the manuscript that addresses the points raised during the review process.

==============================

I’d like to thank authors’ efforts & and endeavour, but I think there is still a remaining concern. To speed up, but also to avoid another invitation of new reviewers, I’d highly recommend the authors to carefully check and address them.

As noticed, the authors have addressed almost everything raised by the reviewers, but some of them did not report or get back for another round. In this sense, remaining aspects (this is an overall concern) are presented here compiling the unsolved issues.

**Responses to reviewers’ Comments**

**Reviewers#3**

Overall, the authors need to better work on Introduction. There is a lack of in-depth debate of the rationale and background of your study. I do not think a simple paragraph would be enough to cover “what’s new” and “what the literature has been saying – on the language’s theories perspective. I am not sure if this was misinterpreted, but the Reviewer requested an extension of Introduction, not only one-two new sentences. This could be considered as a not-so-quick process.

**Introduction:** The Introduction has been modified, where an in-depth analysis has been added (kindly see pages 4- 8). Please have a look at the Introduction section.

**Literature Review:** The authors have also added a sub-section to the literate review, which reviewed the previous studies on wh-questions in Mehri. Kindly see the first sub-section on literature review (pages 8- 11).

After this, the reviewer raised some questions and the authors promptly addressed them. Nevertheless, this should be placed in text (as amendments, i.e. as updated sentences or paragraphs covering these gaps; this should not be written in the same way as the authors placed to the reviewer, but as plain text).

All the answers to the reviewers’ comments and questions have now been placed in the text of the paper. The authors did not a leave a single question or comment without placing its answer in the text. Please have a look at the text of the paper. There are two attached files: one shows the paper with track changes (where modifications and changes have been incorporated in the text in RED) and the other one is a fair copy of the paper after incorporating all the required changes into the text).

Kindly see the following pages to trace the changes and modifications:

1. Pages 26 to 29
2. Page 27 (Endnote iii)
3. Page 33
4. Pages 35 to 36
5. Page 38 (Endnote iv)
6. Pages 38 to 40

Also, two endnotes have been added to the text in response to the reviewers’ comments: endnote iii on page 27 and endnote iv on page 38. These endnotes are placed in the end of the paper, immediately after references (kindly see page 55).

**Reviewer #1**

Finally, I think that would be fine if the authors could address the concerns on stars and/or nomenclature. Although I understand the authors’ point that this won’t be correct due to copyright etc. this can be placed as Sup. File with a sentence “adapted from…” or something similar. This would be really interesting for other researchers and readers too.

Concerning the stars and/ or nomenclature pointed out by one of the reviewers, the authors have added “adopted from (Rizzi, 1997, p. 297) to the clause structure of Rizzi on page 17, placed in the text of the paper. They have also added a Sup. File for this issue upon the request of the reviewer. This is also shown below.

**Figure 4. Tree structure of Rizzi's [1997] Split-CP Hypothesis (adopted from Rizzi, 1997, p. 297)**

Note: This clause structure is on page 23 (in the text of the paper).
